# Supplementary material for: Independently evolved pollution resistance in four killifish populations is largely explained by few variants of large effect
Source: Evol Appl. 2024 Jan 29;17(1):e13648. doi: 10.1111/eva.13648 (PMC10824703; doi:10.1111/eva.13648)
Supplement: Supplementary file 1 — Appendix S1 [file EVA-17-e13648-s001.docx]

| Population | Plate | Plate Well | Individual | Day 10 Phenotype rating | Family | index |
| --- | --- | --- | --- | --- | --- | --- |
| Newark | 105 | A01 | 11155 | 5 | 1413 | ACAAGCTA |
| Newark | 105 | B01 | 11163 | 0 | 1413 | AACAACCA |
| Newark | 105 | C01 | 11169 | 5 | 1413 | AGATCGCA |
| Newark | 105 | D01 | 11170 | 0 | 1413 | CCGAAGTA |
| Newark | 105 | E01 | 11172 | 1 | 1413 | GATAGACA |
| Newark | 105 | F01 | 11175 | 0 | 1413 | TATCAGCA |
| Newark | 105 | G01 | 11179 | 4 | 1413 | AATGTTGC |
| Newark | 105 | H01 | 11180 | 0 | 1413 | CCATCCTC |
| Newark | 105 | A02 | 11184 | 0 | 1413 | AAACATCG |
| Newark | 105 | B02 | 11185 | 0 | 1413 | AACCGAGA |
| Newark | 105 | C02 | 11186 | 5 | 1413 | AGCAGGAA |
| Newark | 105 | D02 | 11189 | 0 | 1413 | CCGTGAGA |
| Newark | 105 | E02 | 11191 | 0 | 1413 | GCCACATA |
| Newark | 105 | F02 | 11193 | 4 | 1413 | TCCGTCTA |
| Newark | 105 | G02 | 11196 | 4 | 1413 | ACACTGAC |
| Newark | 105 | H02 | 11197 | 0 | 1413 | CCGACAAC |
| Newark | 105 | A03 | 11201 | 0 | 1413 | ACATTGGC |
| Newark | 105 | B03 | 11204 | 4 | 1413 | AACGCTTA |
| Newark | 105 | C03 | 11206 | 0 | 1413 | AGTCACTA |
| Newark | 105 | D03 | 11209 | 5 | 1413 | CCTCCTGA |
| Newark | 105 | E03 | 11211 | 1 | 1413 | GCGAGTAA |
| Newark | 105 | F03 | 11213 | 4 | 1413 | TCTTCACA |
| Newark | 105 | G03 | 11214 | 5 | 1413 | ACAGATTC |
| Newark | 105 | H03 | 11218 | 5 | 1413 | AGTCAAGC |
| Newark | 105 | A04 | 11220 | 0 | 1413 | ACCACTGT |
| Newark | 105 | B04 | 11225 | 0 | 1413 | AAGACGGA |
| Newark | 105 | C04 | 11228 | 5 | 1413 | ATCCTGTA |
| Newark | 105 | D04 | 11232 | 5 | 1413 | CGAACTTA |
| Newark | 105 | E04 | 11233 | 0 | 1413 | GCTAACGA |
| Newark | 105 | F04 | 11234 | 1 | 1413 | TGAAGAGA |
| Newark | 105 | G04 | 11235 | 0 | 1413 | AGATGTAC |
| Newark | 105 | H04 | 11237 | 5 | 1413 | CCTCTATC |
| Newark | 105 | A05 | 11240 | 0 | 1413 | AACGTGAT |
| Newark | 105 | B05 | 11244 | 5 | 1413 | AAGGTACA |
| Newark | 105 | C05 | 11250 | 5 | 1413 | ATTGAGGA |
| Newark | 105 | D05 | 11252 | 5 | 1413 | CGACTGGA |
| Newark | 105 | E05 | 11254 | 4 | 1413 | GCTCGGTA |
| Newark | 105 | F05 | 11257 | 0 | 1413 | TGGAACAA |
| Newark | 105 | G05 | 11258 | 4 | 1413 | AGCACCTC |
| Newark | 105 | H05 | 11266 | 5 | 1413 | CGACACAC |
| Newark | 105 | A06 | 11274 | 0 | 1413 | CGCTGATC |
| Newark | 105 | B06 | 11284 | 0 | 1413 | ACACAGAA |
| Newark | 105 | C06 | 11285 | 0 | 1413 | CAACCACA |
| Newark | 105 | D06 | 11286 | 5 | 1413 | CGCATACA |
| Newark | 105 | E06 | 11291 | 4 | 1413 | GGAGAACA |
| Newark | 105 | F06 | 11292 | 0 | 1413 | TGGCTTCA |
| Newark | 105 | G06 | 11293 | 5 | 1413 | AGCCATGC |
| Newark | 105 | H06 | 11296 | 0 | 1413 | CGGATTGC |
| Newark | 105 | A07 | 11298 | 5 | 1413 | CAGATCTG |
| Newark | 105 | B07 | 11299 | 4 | 1413 | ACAGCAGA |
| Newark | 105 | C07 | 11300 | 0 | 1413 | CAAGACTA |
| Newark | 105 | D07 | 11302 | 4 | 1413 | CTCAATGA |
| Newark | 105 | E07 | 11303 | 5 | 1413 | GGTGCGAA |
| Newark | 105 | F07 | 11305 | 5 | 1413 | TGGTGGTA |
| Newark | 105 | G07 | 11308 | 1 | 1413 | AGGCTAAC |
| Newark | 105 | H07 | 11309 | 5 | 1413 | CTAAGGTC |
| Newark | 105 | A08 | 11310 | 5 | 1413 | ATGCCTAA |
| Newark | 105 | B08 | 11311 | 0 | 1413 | ACCTCCAA |
| Newark | 105 | C08 | 11313 | 5 | 1413 | CAATGGAA |
| Newark | 105 | D08 | 11320 | 4 | 1413 | CTGAGCCA |
| Newark | 105 | E08 | 11323 | 5 | 1413 | GTACGCAA |
| Newark | 105 | F08 | 11328 | 0 | 1413 | TTCACGCA |
| Newark | 105 | G08 | 11329 | 0 | 1413 | ATAGCGAC |
| Newark | 105 | H08 | 11331 | 0 | 1413 | GAACAGGC |
| Newark | 105 | A09 | 11332 | 0 | 1413 | AACGAACG |
| Newark | 105 | B09 | 11340 | 0 | 1413 | ACGCTCGA |
| Newark | 105 | C09 | 11342 | 4 | 1413 | CACTTCGA |
| Newark | 105 | D09 | 11344 | 0 | 1413 | CTGGCATA |
| Newark | 105 | E09 | 11348 | 1 | 1413 | GTCGTAGA |
| Newark | 105 | F09 | 11349 | 0 | 1413 | ACACGAGA |
| Newark | 105 | G09 | 11351 | 0 | 1413 | ACGACAAG |
| Newark | 105 | H09 | 11353 | 0 | 1413 | GACAGTGC |
| Newark | 105 | A10 | 11354 | 1 | 1413 | AGTACAAG |
| Newark | 105 | B10 | 11365 | 5 | 1413 | ACGTATCA |
| Newark | 105 | C10 | 11367 | 0 | 1413 | CAGCGTTA |
| Newark | 105 | D10 | 11370 | 5 | 1413 | GAATCTGA |
| Newark | 105 | E10 | 11373 | 1 | 1413 | GTCTGTCA |
| Newark | 105 | F10 | 11374 | 5 | 1413 | AAGAGATC |
| Newark | 105 | G10 | 11375 | 0 | 1413 | ATTGGCTC |
| Newark | 105 | H10 | 11376 | 4 | 1413 | GAGTTAGC |
| Newark | 105 | A11 | 11377 | 5 | 1413 | CATCAAGT |
| Newark | 105 | B11 | 11380 | 5 | 1413 | ACTATGCA |
| Newark | 105 | C11 | 11383 | 4 | 1413 | CATACCAA |
| Newark | 105 | D11 | 11386 | 5 | 1413 | GACTAGTA |
| Newark | 105 | E11 | 11388 | 1 | 1413 | GTGTTCTA |
| Newark | 105 | F11 | 11392 | 5 | 1413 | AAGGACAC |
| Newark | 105 | G11 | 11393 | 0 | 1413 | CAAGGAGC |
| Newark | 105 | H11 | 11395 | 5 | 1413 | GATGAATC |
| Newark | 105 | A12 | 11396 | 0 | 1413 | AGTGGTCA |
| Newark | 105 | B12 | 11398 | 5 | 1413 | AGAGTCAA |
| Newark | 105 | C12 | 11403 | 0 | 1413 | CCAGTTCA |
| Newark | 105 | D12 | 11404 | 4 | 1413 | GAGCTGAA |
| Newark | 105 | E12 | 11407 | 0 | 1413 | TAGGATGA |
| Newark | 105 | F12 | 11409 | 5 | 1413 | AATCCGTC |
| Newark | 105 | G12 | 11410 | 5 | 1413 | CACCTTAC |
| Newark | 105 | H12 | 11412 | 0 | 1413 | GCCAAGAC |
| New.Bedford.Harbor family progenitor | 106 | A01 | NBH Fam 1 ♂ = from BI | #N/A | 812 | ACAAGCTA |
| Bridgeport | 106 | B01 | 2024 | 0 | 812 | AACAACCA |
| Bridgeport | 106 | C01 | 2036 | 0 | 812 | AGATCGCA |
| Bridgeport | 106 | D01 | 2049 | 0 | 812 | CCGAAGTA |
| Bridgeport | 106 | E01 | 2051 | 0 | 812 | GATAGACA |
| Bridgeport | 106 | F01 | 2054 | 3 | 812 | TATCAGCA |
| Bridgeport | 106 | G01 | 2058 | 0 | 812 | AATGTTGC |
| Bridgeport | 106 | H01 | 2060 | 0 | 812 | CCATCCTC |
| Bridgeport | 106 | A02 | 2062 | 0 | 812 | AAACATCG |
| New.Bedford.Harbor family progenitor | 106 | B02 | NBH Fam 1 ♀ = from NBH | #N/A | 812 | AACCGAGA |
| Bridgeport | 106 | C02 | 2065 | 0 | 812 | AGCAGGAA |
| Bridgeport | 106 | D02 | 2066 | 4 | 812 | CCGTGAGA |
| Bridgeport | 106 | E02 | 2072 | 0 | 812 | GCCACATA |
| Bridgeport | 106 | F02 | 2078 | 0 | 812 | TCCGTCTA |
| Bridgeport | 106 | G02 | 2088 | 0 | 812 | ACACTGAC |
| Bridgeport | 106 | H02 | 2091 | 3 | 812 | CCGACAAC |
| Bridgeport | 106 | A03 | 2093 | 0 | 812 | ACATTGGC |
| Bridgeport | 106 | B03 | 2097 | 0 | 812 | AACGCTTA |
| Bridgeport | 106 | C03 | 2103 | 0 | 812 | AGTCACTA |
| Bridgeport | 106 | D03 | 2169 | 3 | 812 | CCTCCTGA |
| Bridgeport | 106 | E03 | 2182 | 4 | 812 | GCGAGTAA |
| Bridgeport | 106 | F03 | 2185 | 3 | 812 | TCTTCACA |
| Bridgeport | 106 | G03 | 2187 | 0 | 812 | ACAGATTC |
| Bridgeport | 106 | H03 | 2189 | 4 | 812 | AGTCAAGC |
| Bridgeport | 106 | A04 | 2192 | 4 | 812 | ACCACTGT |
| Bridgeport | 106 | B04 | 2285 | 3 | 812 | AAGACGGA |
| Bridgeport | 106 | C04 | 2289 | 4 | 812 | ATCCTGTA |
| Bridgeport | 106 | D04 | 2290 | 0 | 812 | CGAACTTA |
| Bridgeport | 106 | E04 | 2293 | 4 | 812 | GCTAACGA |
| Bridgeport | 106 | F04 | 2305 | 0 | 812 | TGAAGAGA |
| Bridgeport | 106 | G04 | 2308 | 4 | 812 | AGATGTAC |
| Bridgeport | 106 | H04 | 2314 | 0 | 812 | CCTCTATC |
| Bridgeport | 106 | A05 | 2316 | 4 | 812 | AACGTGAT |
| Bridgeport | 106 | B05 | 2317 | 0 | 812 | AAGGTACA |
| Bridgeport | 106 | C05 | 2339 | 4 | 812 | ATTGAGGA |
| Bridgeport | 106 | D05 | 2340 | 0 | 812 | CGACTGGA |
| Bridgeport | 106 | E05 | 2344 | 0 | 812 | GCTCGGTA |
| Bridgeport | 106 | F05 | 2345 | 3 | 812 | TGGAACAA |
| Bridgeport | 106 | G05 | 2346 | 0 | 812 | AGCACCTC |
| Bridgeport | 106 | H05 | 2354 | 4 | 812 | CGACACAC |
| Bridgeport | 106 | A06 | 2356 | 3 | 812 | CGCTGATC |
| Bridgeport | 106 | B06 | 2358 | 3 | 812 | ACACAGAA |
| Bridgeport | 106 | C06 | 2403 | 4 | 812 | CAACCACA |
| Bridgeport | 106 | D06 | 2404 | 4 | 812 | CGCATACA |
| Bridgeport | 106 | E06 | 2409 | 0 | 812 | GGAGAACA |
| Bridgeport | 106 | F06 | 2410 | 4 | 812 | TGGCTTCA |
| Bridgeport | 106 | G06 | 2412 | 0 | 812 | AGCCATGC |
| Bridgeport | 106 | H06 | 2413 | 4 | 812 | CGGATTGC |
| Bridgeport | 106 | A07 | 2414 | 4 | 812 | CAGATCTG |
| Bridgeport | 106 | B07 | 2420 | 0 | 812 | ACAGCAGA |
| Bridgeport | 106 | C07 | 2422 | 3 | 812 | CAAGACTA |
| Bridgeport | 106 | D07 | 2427 | 4 | 812 | CTCAATGA |
| Bridgeport | 106 | E07 | 2428 | 0 | 812 | GGTGCGAA |
| Bridgeport | 106 | F07 | 2429 | 4 | 812 | TGGTGGTA |
| Bridgeport | 106 | G07 | 2434 | 0 | 812 | AGGCTAAC |
| Bridgeport | 106 | H07 | 2436 | 0 | 812 | CTAAGGTC |
| Bridgeport | 106 | A08 | 2440 | 0 | 812 | ATGCCTAA |
| Bridgeport | 106 | B08 | 2447 | 0 | 812 | ACCTCCAA |
| Bridgeport | 106 | C08 | 2521 | 0 | 812 | CAATGGAA |
| Bridgeport | 106 | D08 | 2524 | 0 | 812 | CTGAGCCA |
| Bridgeport | 106 | E08 | 2532 | 4 | 812 | GTACGCAA |
| Bridgeport | 106 | F08 | 2534 | 4 | 812 | TTCACGCA |
| Bridgeport | 106 | G08 | 2535 | 0 | 812 | ATAGCGAC |
| Bridgeport | 106 | H08 | 2538 | 4 | 812 | GAACAGGC |
| Bridgeport | 106 | A09 | 2546 | 0 | 812 | AACGAACG |
| Bridgeport | 106 | B09 | 2547 | 4 | 812 | ACGCTCGA |
| Bridgeport | 106 | C09 | 2551 | 4 | 812 | CACTTCGA |
| Bridgeport | 106 | D09 | 2553 | 0 | 812 | CTGGCATA |
| Bridgeport | 106 | E09 | 2554 | 4 | 812 | GTCGTAGA |
| Bridgeport | 106 | F09 | 2555 | 3 | 812 | ACACGAGA |
| Bridgeport | 106 | G09 | 2562 | 3 | 812 | ACGACAAG |
| Bridgeport | 106 | H09 | 2563 | 3 | 812 | GACAGTGC |
| Bridgeport | 106 | A10 | 2566 | 0 | 812 | AGTACAAG |
| Bridgeport | 106 | B10 | 2567 | 0 | 812 | ACGTATCA |
| Bridgeport | 106 | C10 | 2572 | 3 | 812 | CAGCGTTA |
| Bridgeport | 106 | D10 | 2573 | 0 | 812 | GAATCTGA |
| Bridgeport | 106 | E10 | 2575 | 0 | 812 | GTCTGTCA |
| Bridgeport | 106 | F10 | 2576 | 0 | 812 | AAGAGATC |
| Bridgeport | 106 | G10 | 2578 | 3 | 812 | ATTGGCTC |
| Bridgeport | 106 | H10 | 2671 | 4 | 812 | GAGTTAGC |
| Bridgeport | 106 | A11 | 2672 | 0 | 812 | CATCAAGT |
| Bridgeport | 106 | B11 | 2674 | 3 | 812 | ACTATGCA |
| Bridgeport | 106 | C11 | 2676 | 4 | 812 | CATACCAA |
| Bridgeport | 106 | D11 | 2677 | 0 | 812 | GACTAGTA |
| Bridgeport | 106 | E11 | 2678 | 0 | 812 | GTGTTCTA |
| Bridgeport | 106 | F11 | 2681 | 0 | 812 | AAGGACAC |
| Bridgeport | 106 | G11 | 2686 | 4 | 812 | CAAGGAGC |
| Bridgeport | 106 | H11 | 2687 | 4 | 812 | GATGAATC |
| Bridgeport | 106 | A12 | 2688 | 4 | 812 | AGTGGTCA |
| Bridgeport | 106 | B12 | 2695 | 4 | 812 | AGAGTCAA |
| Bridgeport | 106 | C12 | 2697 | 0 | 812 | CCAGTTCA |
| Bridgeport | 106 | D12 | 2699 | 4 | 812 | GAGCTGAA |
| Bridgeport | 106 | E12 | 2700 | 0 | 812 | TAGGATGA |
| Bridgeport | 106 | F12 | 2708 | 4 | 812 | AATCCGTC |
| Bridgeport | 106 | G12 | 2710 | 4 | 812 | CACCTTAC |
| Bridgeport | 106 | H12 | 2719 | 0 | 812 | GCCAAGAC |
| Elizabeth.River | 107 | A01 | 10869 | 5 | 1433 | ACAAGCTA |
| Elizabeth.River | 107 | B01 | 10870 | 1 | 1433 | AACAACCA |
| Elizabeth.River | 107 | C01 | 10871 | 4 | 1433 | AGATCGCA |
| Elizabeth.River | 107 | D01 | 10872 | 0 | 1433 | CCGAAGTA |
| Elizabeth.River | 107 | E01 | 10874 | 5 | 1433 | GATAGACA |
| Elizabeth.River | 107 | F01 | 10876 | 0 | 1433 | TATCAGCA |
| Elizabeth.River | 107 | G01 | 10878 | 4 | 1433 | AATGTTGC |
| Elizabeth.River | 107 | H01 | 10881 | 0 | 1433 | CCATCCTC |
| Elizabeth.River | 107 | A02 | 10882 | 5 | 1433 | AAACATCG |
| Elizabeth.River | 107 | B02 | 10884 | 4 | 1433 | AACCGAGA |
| Elizabeth.River | 107 | C02 | 10885 | 5 | 1433 | AGCAGGAA |
| Elizabeth.River | 107 | D02 | 10921 | 0 | 1433 | CCGTGAGA |
| Elizabeth.River | 107 | E02 | 10923 | 4 | 1433 | GCCACATA |
| Elizabeth.River | 107 | F02 | 10924 | 4 | 1433 | TCCGTCTA |
| Elizabeth.River | 107 | G02 | 10925 | 0 | 1433 | ACACTGAC |
| Elizabeth.River | 107 | H02 | 10926 | 5 | 1433 | CCGACAAC |
| Elizabeth.River | 107 | A03 | 10928 | 5 | 1433 | ACATTGGC |
| Elizabeth.River | 107 | B03 | 10929 | 4 | 1433 | AACGCTTA |
| Elizabeth.River | 107 | C03 | 10930 | 0 | 1433 | AGTCACTA |
| Elizabeth.River | 107 | D03 | 10932 | 4 | 1433 | CCTCCTGA |
| Elizabeth.River | 107 | E03 | 10953 | 4 | 1433 | GCGAGTAA |
| Elizabeth.River | 107 | F03 | 10954 | 0 | 1433 | TCTTCACA |
| Elizabeth.River | 107 | G03 | 10956 | 0 | 1433 | ACAGATTC |
| Elizabeth.River | 107 | H03 | 10960 | 0 | 1433 | AGTCAAGC |
| Elizabeth.River | 107 | A04 | 10961 | 0 | 1433 | ACCACTGT |
| Elizabeth.River | 107 | B04 | 10963 | 5 | 1433 | AAGACGGA |
| Elizabeth.River | 107 | C04 | 10964 | 4 | 1433 | ATCCTGTA |
| Elizabeth.River | 107 | D04 | 10965 | 1 | 1433 | CGAACTTA |
| Elizabeth.River | 107 | E04 | 10967 | 4 | 1433 | GCTAACGA |
| Elizabeth.River | 107 | F04 | 10968 | 0 | 1433 | TGAAGAGA |
| Elizabeth.River | 107 | G04 | 10969 | 5 | 1433 | AGATGTAC |
| Elizabeth.River | 107 | H04 | 10970 | 0 | 1433 | CCTCTATC |
| Elizabeth.River | 107 | A05 | 10971 | 4 | 1433 | AACGTGAT |
| Elizabeth.River | 107 | B05 | 10972 | 1 | 1433 | AAGGTACA |
| Elizabeth.River | 107 | C05 | 10973 | 4 | 1433 | ATTGAGGA |
| Elizabeth.River | 107 | D05 | 10974 | 5 | 1433 | CGACTGGA |
| Elizabeth.River | 107 | E05 | 10977 | 0 | 1433 | GCTCGGTA |
| Elizabeth.River | 107 | F05 | 10978 | 0 | 1433 | TGGAACAA |
| Elizabeth.River | 107 | G05 | 10979 | 0 | 1433 | AGCACCTC |
| Elizabeth.River | 107 | H05 | 10980 | 5 | 1433 | CGACACAC |
| Elizabeth.River | 107 | A06 | 10981 | 5 | 1433 | CGCTGATC |
| Elizabeth.River | 107 | B06 | 10982 | 5 | 1433 | ACACAGAA |
| Elizabeth.River | 107 | C06 | 10983 | 0 | 1433 | CAACCACA |
| Elizabeth.River | 107 | D06 | 10984 | 0 | 1433 | CGCATACA |
| Elizabeth.River | 107 | E06 | 10986 | 5 | 1433 | GGAGAACA |
| Elizabeth.River | 107 | F06 | 10987 | 1 | 1433 | TGGCTTCA |
| Elizabeth.River | 107 | G06 | 10988 | 0 | 1433 | AGCCATGC |
| Elizabeth.River | 107 | H06 | 10989 | 5 | 1433 | CGGATTGC |
| Elizabeth.River | 107 | A07 | 10990 | 4 | 1433 | CAGATCTG |
| Elizabeth.River | 107 | B07 | 10991 | 0 | 1433 | ACAGCAGA |
| Elizabeth.River | 107 | C07 | 10993 | 0 | 1433 | CAAGACTA |
| Elizabeth.River | 107 | D07 | 10994 | 0 | 1433 | CTCAATGA |
| Elizabeth.River | 107 | E07 | 10997 | 5 | 1433 | GGTGCGAA |
| Elizabeth.River | 107 | F07 | 10998 | 5 | 1433 | TGGTGGTA |
| Elizabeth.River | 107 | G07 | 10999 | 4 | 1433 | AGGCTAAC |
| Elizabeth.River | 107 | H07 | 11000 | 1 | 1433 | CTAAGGTC |
| Elizabeth.River | 107 | A08 | 11001 | 1 | 1433 | ATGCCTAA |
| Elizabeth.River | 107 | B08 | 11002 | 5 | 1433 | ACCTCCAA |
| Elizabeth.River | 107 | C08 | 11004 | 4 | 1433 | CAATGGAA |
| Elizabeth.River | 107 | D08 | 11010 | 4 | 1433 | CTGAGCCA |
| Elizabeth.River | 107 | E08 | 11011 | 1 | 1433 | GTACGCAA |
| Elizabeth.River | 107 | F08 | 11102 | 0 | 1433 | TTCACGCA |
| Elizabeth.River | 107 | G08 | 11103 | 5 | 1433 | ATAGCGAC |
| Elizabeth.River | 107 | H08 | 11104 | 5 | 1433 | GAACAGGC |
| Elizabeth.River | 107 | A09 | 11105 | 4 | 1433 | AACGAACG |
| Elizabeth.River | 107 | B09 | 11108 | 1 | 1433 | ACGCTCGA |
| Elizabeth.River | 107 | C09 | 11111 | 0 | 1433 | CACTTCGA |
| Elizabeth.River | 107 | D09 | 11112 | 0 | 1433 | CTGGCATA |
| Elizabeth.River | 107 | E09 | 11114 | 0 | 1433 | GTCGTAGA |
| Elizabeth.River | 107 | F09 | 11115 | 5 | 1433 | ACACGAGA |
| Elizabeth.River | 107 | G09 | 11116 | 5 | 1433 | ACGACAAG |
| Elizabeth.River | 107 | H09 | 11575 | 1 | 1433 | GACAGTGC |
| Elizabeth.River | 107 | A10 | 11576 | 1 | 1433 | AGTACAAG |
| Elizabeth.River | 107 | B10 | 11577 | 5 | 1433 | ACGTATCA |
| Elizabeth.River | 107 | C10 | 11578 | 1 | 1433 | CAGCGTTA |
| Elizabeth.River | 107 | D10 | 11579 | 5 | 1433 | GAATCTGA |
| Elizabeth.River | 107 | E10 | 11580 | 4 | 1433 | GTCTGTCA |
| Elizabeth.River | 107 | F10 | 11581 | 0 | 1433 | AAGAGATC |
| Elizabeth.River | 107 | G10 | 11583 | 5 | 1433 | ATTGGCTC |
| Elizabeth.River | 107 | H10 | 11584 | 5 | 1433 | GAGTTAGC |
| Elizabeth.River | 107 | A11 | 11585 | 0 | 1433 | CATCAAGT |
| Elizabeth.River | 107 | B11 | 11586 | 5 | 1433 | ACTATGCA |
| Elizabeth.River | 107 | C11 | 11587 | 5 | 1433 | CATACCAA |
| Elizabeth.River | 107 | D11 | 11588 | 0 | 1433 | GACTAGTA |
| Elizabeth.River | 107 | E11 | 11591 | 5 | 1433 | GTGTTCTA |
| Elizabeth.River | 107 | F11 | 11592 | 5 | 1433 | AAGGACAC |
| Elizabeth.River | 107 | G11 | 11593 | 5 | 1433 | CAAGGAGC |
| Elizabeth.River | 107 | H11 | 11594 | 0 | 1433 | GATGAATC |
| Bridgeport family progenitor | 107 | A12 | BP Fam 8 ♀ = from BI | #N/A | 812 | AGTGGTCA |
| Bridgeport family progenitor | 107 | B12 | BP Fam 8 ♂ = from BP | #N/A | 812 | AGAGTCAA |
| Bridgeport family progenitor | 107 | C12 | BP Fam 1 ♀ = from BI | #N/A | 812 | CCAGTTCA |
| Bridgeport family progenitor | 107 | D12 | BP Fam 1 ♂ = from BP | #N/A | 812 | GAGCTGAA |
| Elizabeth.River family progenitor | 107 | E12 | 1124 ER ♀ = from ER | #N/A | 1433 | TAGGATGA |
| Elizabeth.River family progenitor | 107 | F12 | 1124 BI ♂ = from BI | #N/A | 1433 | AATCCGTC |
| Newark family progenitor | 107 | G12 | 911 New ♂ = from New | #N/A | 1413 | CACCTTAC |
| Newark family progenitor | 107 | H12 | 911 New ♀ = from BI | #N/A | 1413 | GCCAAGAC |
| New.Bedford.Harbor | 1 | H01 | 5528 | 0 | 1105 | CCATCCTC |
| New.Bedford.Harbor | 1 | G01 | 5560 | 0 | 1105 | AATGTTGC |
| New.Bedford.Harbor | 1 | F01 | 5563 | 0 | 1105 | TATCAGCA |
| New.Bedford.Harbor | 1 | E01 | 5573 | 0 | 1105 | GATAGACA |
| New.Bedford.Harbor | 1 | D01 | 5574 | 0 | 1105 | CCGAAGTA |
| New.Bedford.Harbor | 1 | C01 | 5577 | 0 | 1105 | AGATCGCA |
| New.Bedford.Harbor | 1 | B01 | 5584 | 0 | 1105 | AACAACCA |
| New.Bedford.Harbor | 1 | A01 | 5595 | 0 | 1105 | ACAAGCTA |
| New.Bedford.Harbor | 1 | H02 | 5596 | 0 | 1105 | CCGACAAC |
| New.Bedford.Harbor | 1 | G02 | 5602 | 0 | 1105 | ACACTGAC |
| New.Bedford.Harbor | 1 | F02 | 5607 | 0 | 1105 | TCCGTCTA |
| New.Bedford.Harbor | 1 | E02 | 5610 | 0 | 1105 | GCCACATA |
| New.Bedford.Harbor | 1 | D02 | 5619 | 0 | 1105 | CCGTGAGA |
| New.Bedford.Harbor | 1 | C02 | 5622 | 0 | 1105 | AGCAGGAA |
| New.Bedford.Harbor | 1 | B02 | 5627 | 0 | 1105 | AACCGAGA |
| New.Bedford.Harbor | 1 | A02 | 5646 | 0 | 1105 | AAACATCG |
| New.Bedford.Harbor | 1 | H03 | 5651 | 0 | 1105 | AGTCAAGC |
| New.Bedford.Harbor | 1 | G03 | 5749 | 0 | 1105 | ACAGATTC |
| New.Bedford.Harbor | 1 | F03 | 5754 | 0 | 1105 | TCTTCACA |
| New.Bedford.Harbor | 1 | E03 | 5813 | 0 | 1105 | GCGAGTAA |
| New.Bedford.Harbor | 1 | D03 | 5820 | 0 | 1105 | CCTCCTGA |
| New.Bedford.Harbor | 1 | C03 | 5830 | 0 | 1105 | AGTCACTA |
| New.Bedford.Harbor | 1 | B03 | 5834 | 0 | 1105 | AACGCTTA |
| New.Bedford.Harbor | 1 | A03 | 6114 | 0 | 1105 | ACATTGGC |
| New.Bedford.Harbor | 1 | H04 | 6125 | 0 | 1105 | CCTCTATC |
| New.Bedford.Harbor | 1 | G04 | 6152 | 0 | 1105 | AGATGTAC |
| New.Bedford.Harbor | 1 | F04 | 6154 | 0 | 1105 | TGAAGAGA |
| New.Bedford.Harbor | 1 | E04 | 6156 | 0 | 1105 | GCTAACGA |
| New.Bedford.Harbor | 1 | D04 | 6157 | 0 | 1105 | CGAACTTA |
| New.Bedford.Harbor | 1 | C04 | 6160 | 0 | 1105 | ATCCTGTA |
| New.Bedford.Harbor | 1 | B04 | 6161 | 0 | 1105 | AAGACGGA |
| New.Bedford.Harbor | 1 | A04 | 6173 | 0 | 1105 | ACCACTGT |
| New.Bedford.Harbor | 1 | H05 | 6174 | 0 | 1105 | CGACACAC |
| New.Bedford.Harbor | 1 | G05 | 6177 | 0 | 1105 | AGCACCTC |
| New.Bedford.Harbor | 1 | F05 | 6185 | 0 | 1105 | TGGAACAA |
| New.Bedford.Harbor | 1 | E05 | 5525 | 1 | 1105 | GCTCGGTA |
| New.Bedford.Harbor | 1 | D05 | 5547 | 1 | 1105 | CGACTGGA |
| New.Bedford.Harbor | 1 | C05 | 5608 | 1 | 1105 | ATTGAGGA |
| New.Bedford.Harbor | 1 | B05 | 5638 | 1 | 1105 | AAGGTACA |
| New.Bedford.Harbor | 1 | A05 | 5639 | 1 | 1105 | AACGTGAT |
| New.Bedford.Harbor | 1 | H06 | 5650 | 1 | 1105 | CGGATTGC |
| New.Bedford.Harbor | 1 | G06 | 5739 | 1 | 1105 | AGCCATGC |
| New.Bedford.Harbor | 1 | F06 | 5949 | 1 | 1105 | TGGCTTCA |
| New.Bedford.Harbor | 1 | E06 | 5956 | 1 | 1105 | GGAGAACA |
| New.Bedford.Harbor | 1 | D06 | 5958 | 1 | 1105 | CGCATACA |
| New.Bedford.Harbor | 1 | C06 | 6127 | 1 | 1105 | CAACCACA |
| New.Bedford.Harbor | 1 | B06 | 6140 | 1 | 1105 | ACACAGAA |
| New.Bedford.Harbor | 1 | A06 | 6155 | 1 | 1105 | CGCTGATC |
| New.Bedford.Harbor | 1 | H07 | 5647 | 4 | 1105 | CTAAGGTC |
| New.Bedford.Harbor | 1 | G07 | 5648 | 4 | 1105 | AGGCTAAC |
| New.Bedford.Harbor | 1 | F07 | 6137 | 4 | 1105 | TGGTGGTA |
| New.Bedford.Harbor | 1 | E07 | 6138 | 4 | 1105 | GGTGCGAA |
| New.Bedford.Harbor | 1 | D07 | 6149 | 4 | 1105 | CTCAATGA |
| New.Bedford.Harbor | 1 | C07 | 6153 | 4 | 1105 | CAAGACTA |
| New.Bedford.Harbor | 1 | B07 | 6165 | 4 | 1105 | ACAGCAGA |
| New.Bedford.Harbor | 1 | A07 | 6171 | 4 | 1105 | CAGATCTG |
| New.Bedford.Harbor | 1 | H08 | 6175 | 4 | 1105 | GAACAGGC |
| New.Bedford.Harbor | 1 | G08 | 6176 | 4 | 1105 | ATAGCGAC |
| New.Bedford.Harbor | 1 | F08 | 6183 | 4 | 1105 | TTCACGCA |
| New.Bedford.Harbor | 1 | E08 | 6184 | 4 | 1105 | GTACGCAA |
| New.Bedford.Harbor | 1 | D08 | 5529 | 5 | 1105 | CTGAGCCA |
| New.Bedford.Harbor | 1 | C08 | 5537 | 5 | 1105 | CAATGGAA |
| New.Bedford.Harbor | 1 | B08 | 5539 | 5 | 1105 | ACCTCCAA |
| New.Bedford.Harbor | 1 | A08 | 5540 | 5 | 1105 | ATGCCTAA |
| New.Bedford.Harbor | 1 | H09 | 5541 | 5 | 1105 | GACAGTGC |
| New.Bedford.Harbor | 1 | G09 | 5543 | 5 | 1105 | ACGACAAG |
| New.Bedford.Harbor | 1 | F09 | 5549 | 5 | 1105 | ACACGAGA |
| New.Bedford.Harbor | 1 | E09 | 5550 | 5 | 1105 | GTCGTAGA |
| New.Bedford.Harbor | 1 | D09 | 5553 | 5 | 1105 | CTGGCATA |
| New.Bedford.Harbor | 1 | C09 | 5555 | 5 | 1105 | CACTTCGA |
| New.Bedford.Harbor | 1 | B09 | 5557 | 5 | 1105 | ACGCTCGA |
| New.Bedford.Harbor | 1 | A09 | 5564 | 5 | 1105 | AACGAACG |
| New.Bedford.Harbor | 1 | H10 | 5578 | 5 | 1105 | GAGTTAGC |
| New.Bedford.Harbor | 1 | G10 | 5585 | 5 | 1105 | ATTGGCTC |
| New.Bedford.Harbor | 1 | F10 | 5586 | 5 | 1105 | AAGAGATC |
| New.Bedford.Harbor | 1 | E10 | 5588 | 5 | 1105 | GTCTGTCA |
| New.Bedford.Harbor | 1 | D10 | 5590 | 5 | 1105 | GAATCTGA |
| New.Bedford.Harbor | 1 | C10 | 5604 | 5 | 1105 | CAGCGTTA |
| New.Bedford.Harbor | 1 | B10 | 5605 | 5 | 1105 | ACGTATCA |
| New.Bedford.Harbor | 1 | A10 | 5615 | 5 | 1105 | AGTACAAG |
| New.Bedford.Harbor | 1 | H11 | 5616 | 5 | 1105 | GATGAATC |
| New.Bedford.Harbor | 1 | G11 | 5618 | 5 | 1105 | CAAGGAGC |
| New.Bedford.Harbor | 1 | F11 | 5630 | 5 | 1105 | AAGGACAC |
| New.Bedford.Harbor | 1 | E11 | 5635 | 5 | 1105 | GTGTTCTA |
| New.Bedford.Harbor | 1 | D11 | 5641 | 5 | 1105 | GACTAGTA |
| New.Bedford.Harbor | 1 | C11 | 5644 | 5 | 1105 | CATACCAA |
| New.Bedford.Harbor | 1 | B11 | 5645 | 5 | 1105 | ACTATGCA |
| New.Bedford.Harbor | 1 | A11 | 5735 | 5 | 1105 | CATCAAGT |
| New.Bedford.Harbor | 1 | H12 | 5831 | 5 | 1105 | GCCAAGAC |
| New.Bedford.Harbor | 1 | G12 | 5832 | 5 | 1105 | CACCTTAC |
| New.Bedford.Harbor | 1 | F12 | 5951 | 5 | 1105 | AATCCGTC |
| New.Bedford.Harbor | 1 | E12 | 5957 | 5 | 1105 | TAGGATGA |
| New.Bedford.Harbor | 1 | D12 | 6126 | 5 | 1105 | GAGCTGAA |
| New.Bedford.Harbor | 1 | C12 | 6130 | 5 | 1105 | CCAGTTCA |
| New.Bedford.Harbor | 1 | B12 | 6151 | 5 | 1105 | AGAGTCAA |
| New.Bedford.Harbor | 1 | A12 | 6178 | 5 | 1105 | AGTGGTCA |
