# Supplementary figures and images for: Independently evolved pollution resistance in four killifish populations is largely explained by few variants of large effect
Source: Evol Appl. 2024 Jan 29;17(1):e13648. doi: 10.1111/eva.13648 (PMC10824703; doi:10.1111/eva.13648)

# AHR signaling pathway

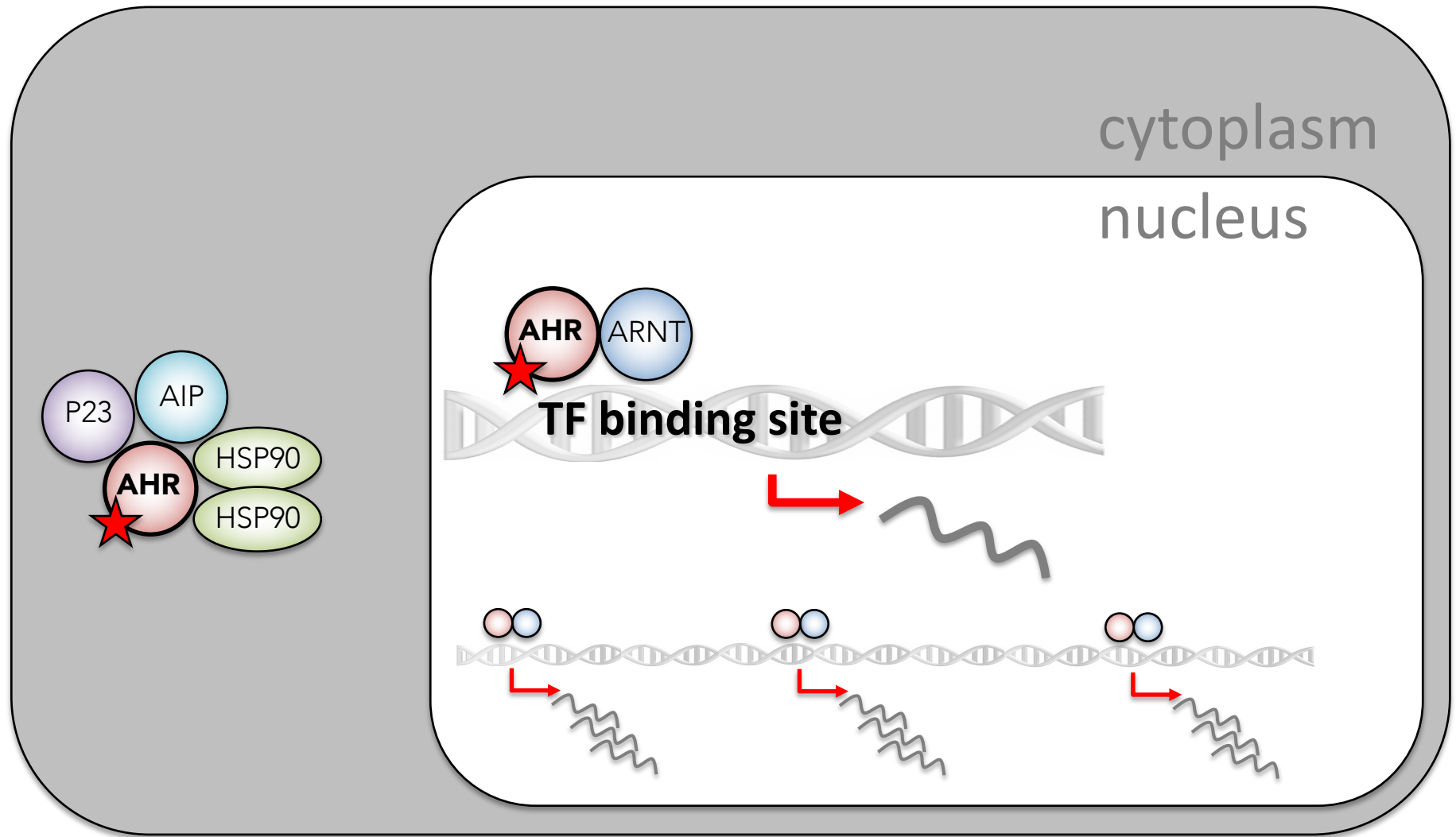

★ AHR ligand: e.g., dioxin, PCB, PAH

Supplement: Supplementary file 4 — Figure S1 [file EVA-17-e13648-s013.pdf]

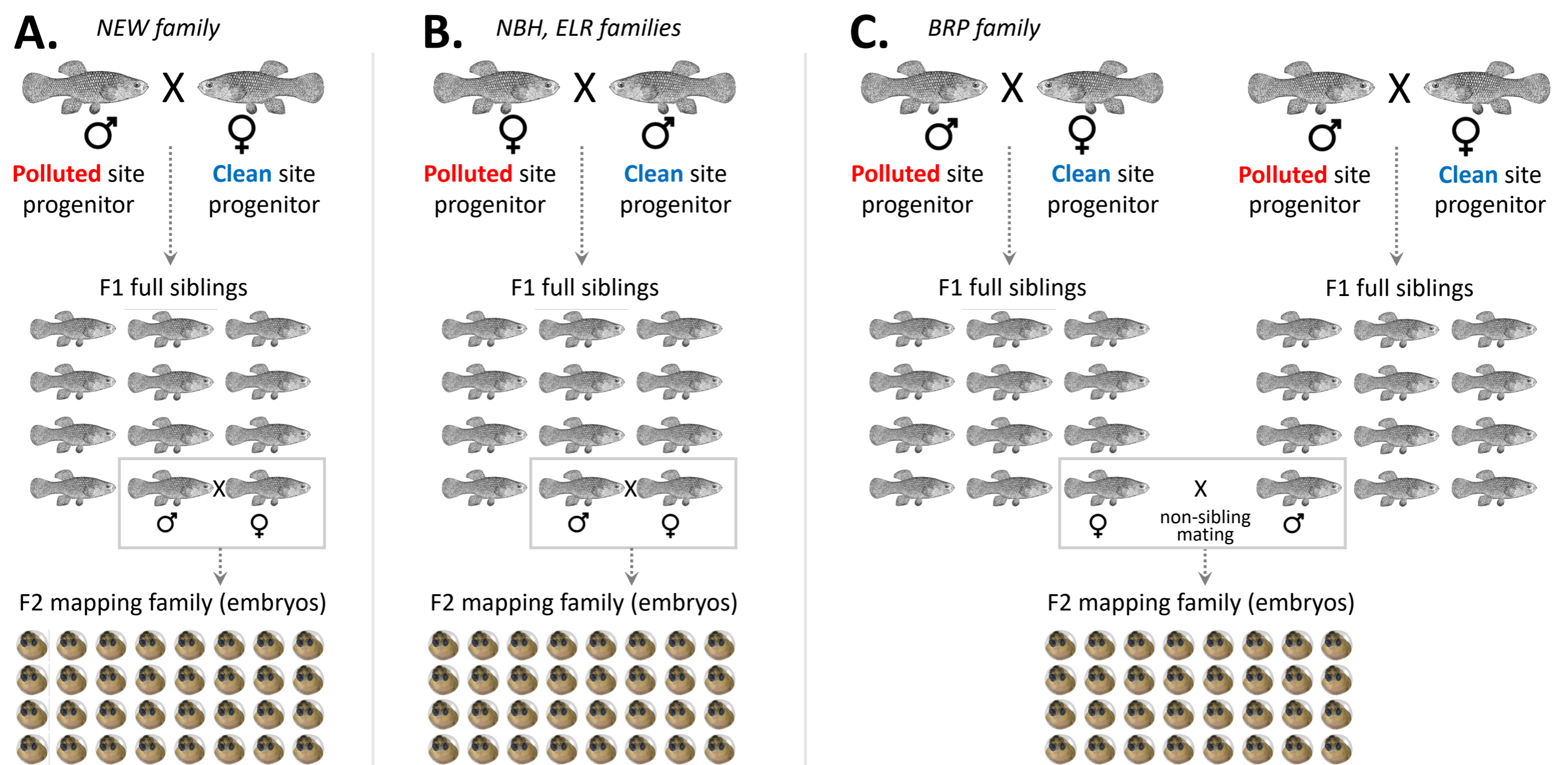

Supplement: Supplementary file 5 — Figure S2 [file EVA-17-e13648-s006.pdf]

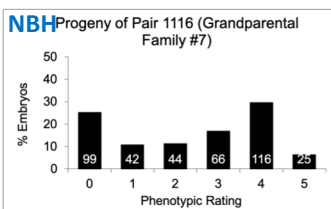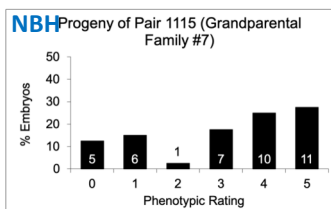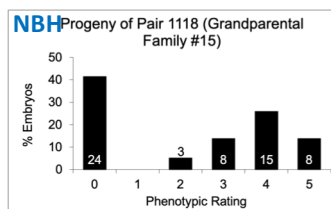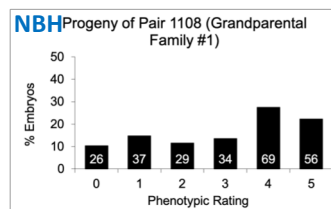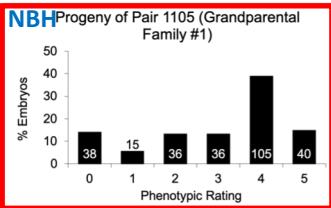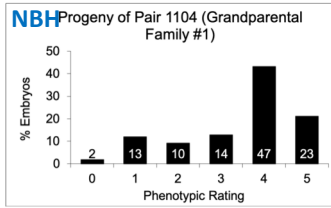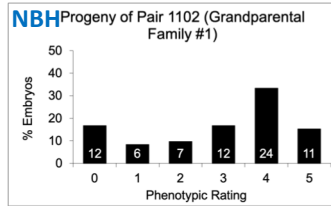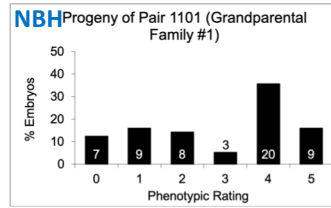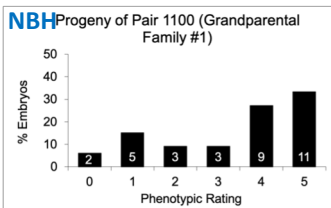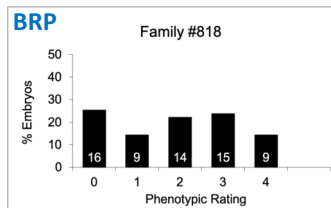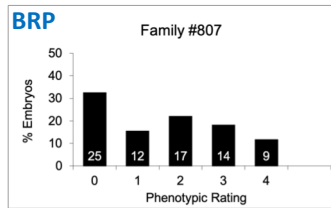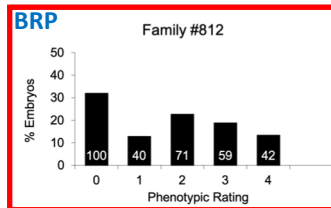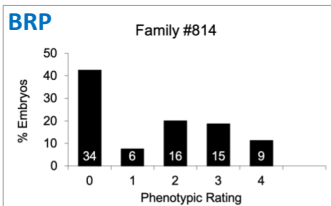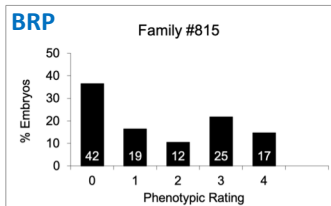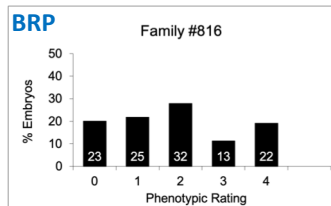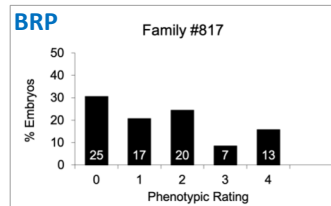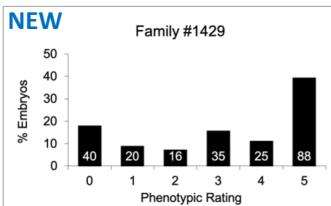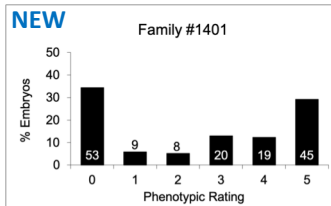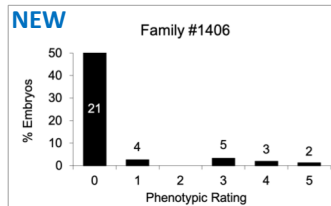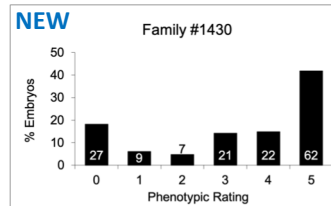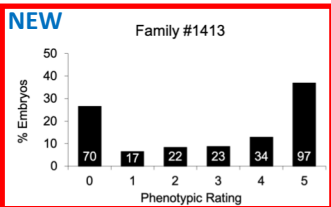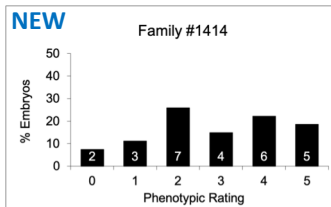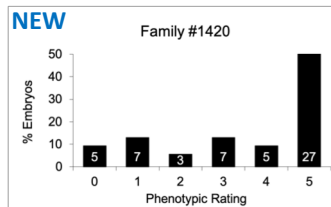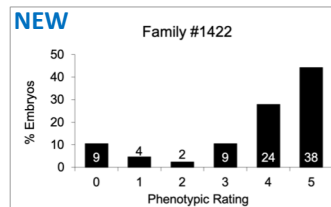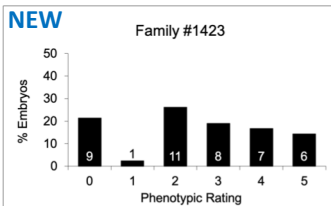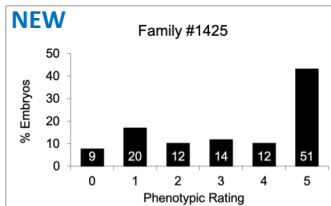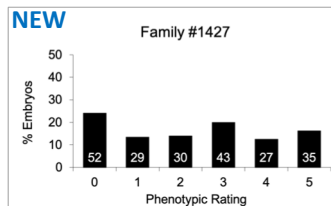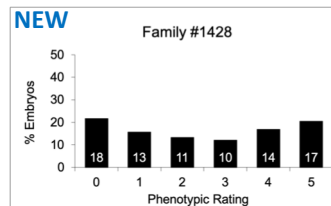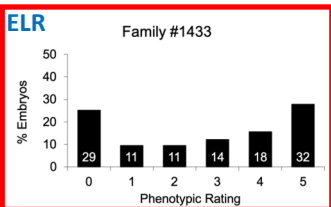

Supplement: Supplementary file 6 — Figure S3 [file EVA-17-e13648-s005.pdf]

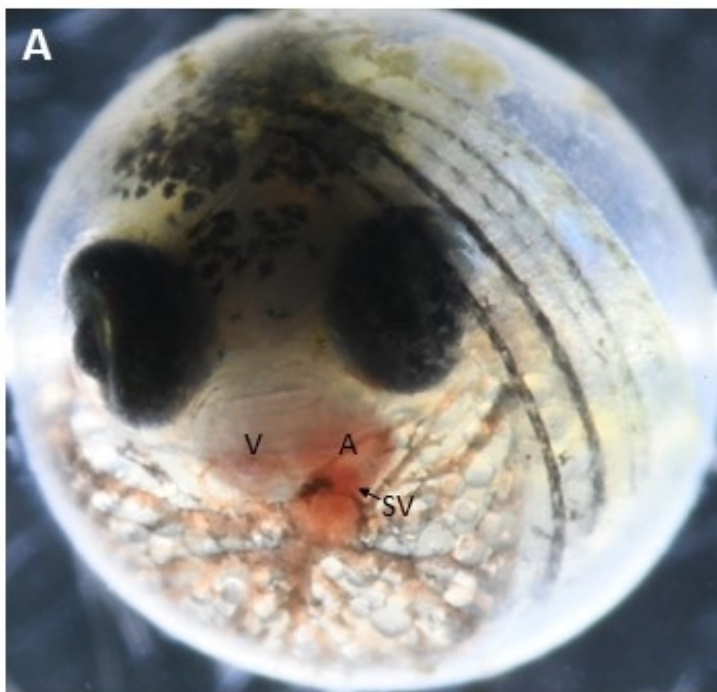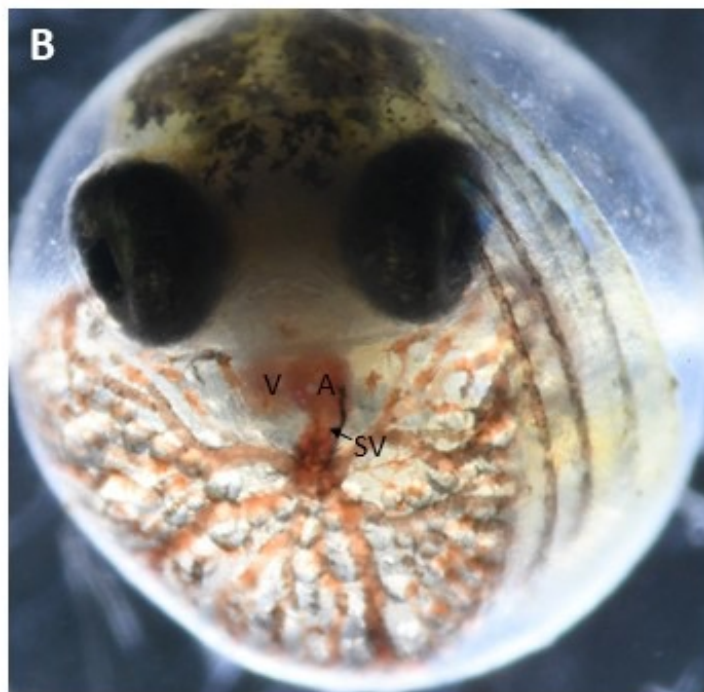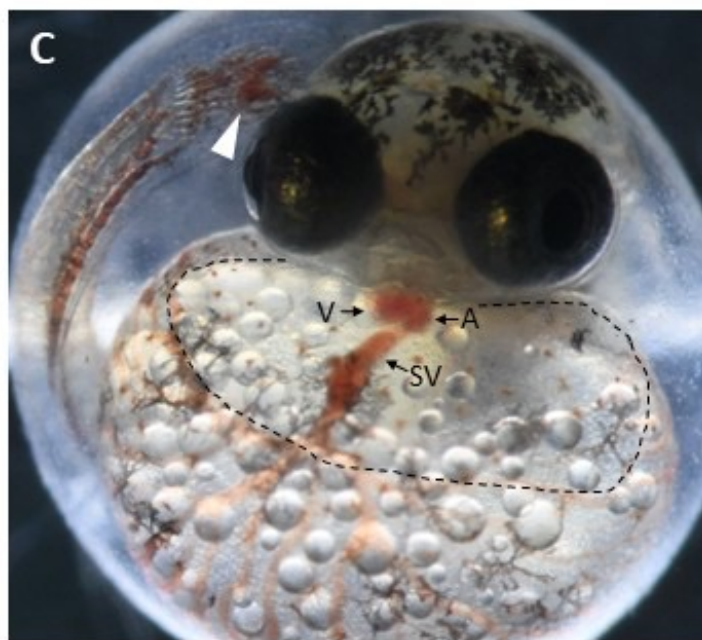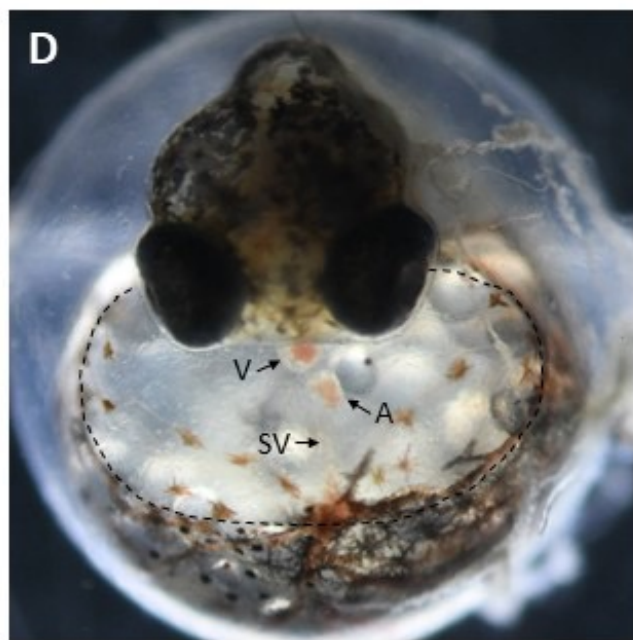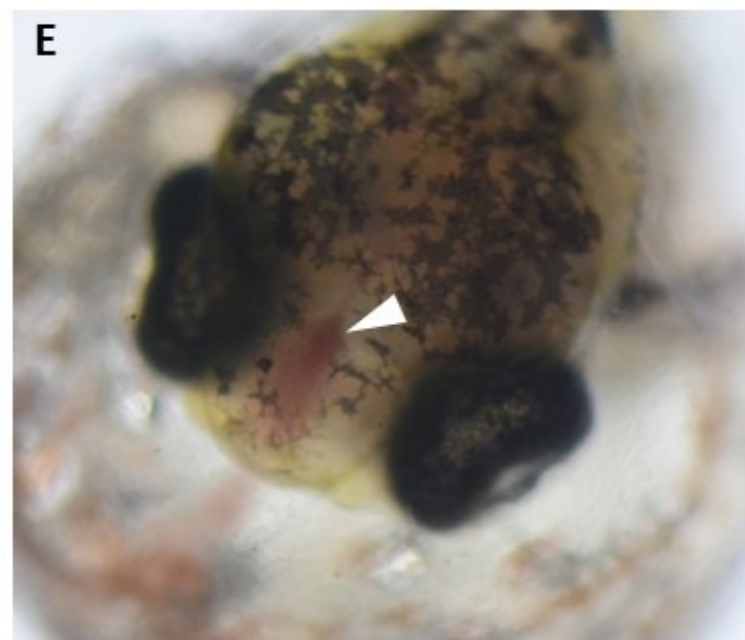

Supplement: Supplementary file 7 — Figure S4 [file EVA-17-e13648-s012.pdf]

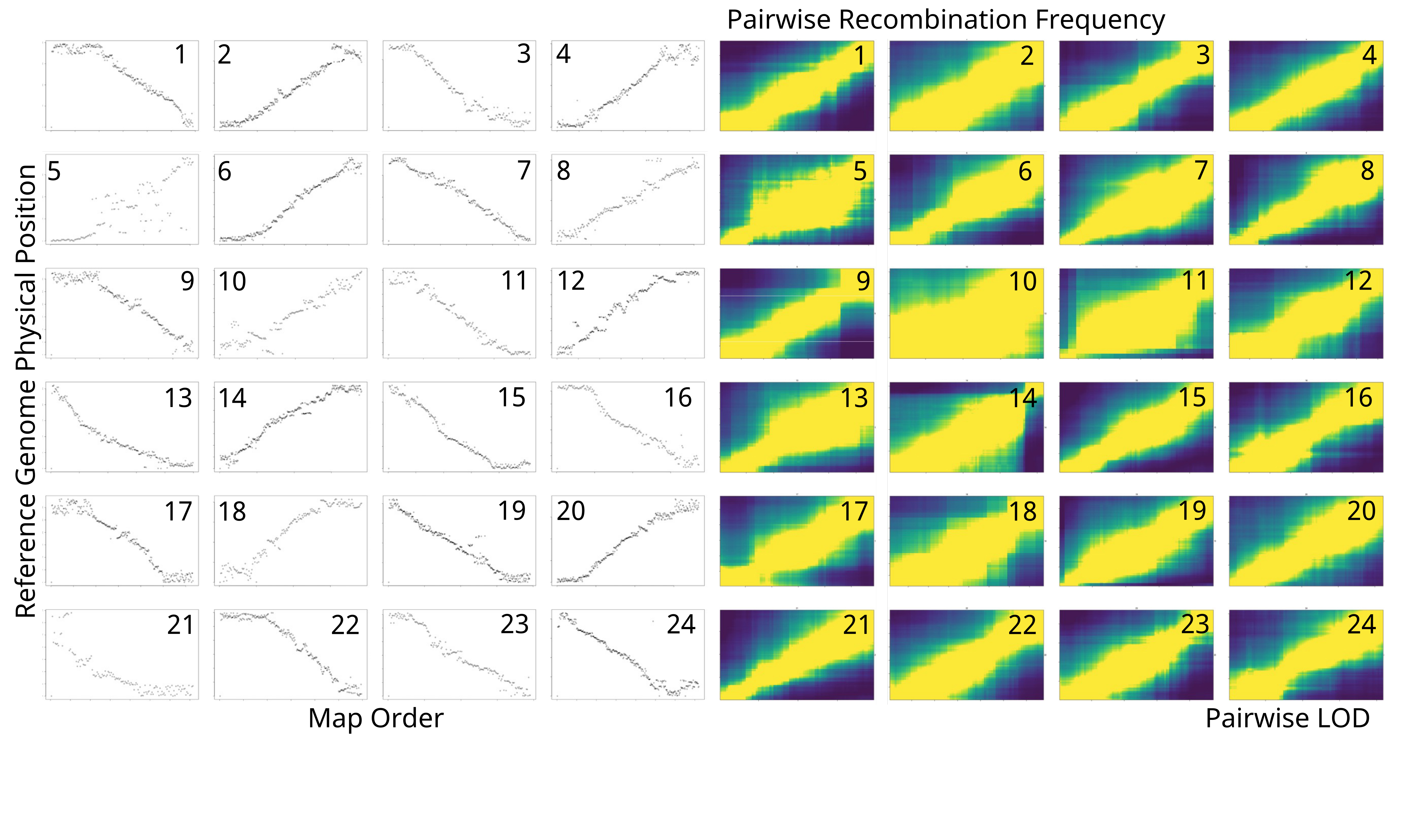

Supplement: Supplementary file 9 — Figure S6 [file EVA-17-e13648-s010.jpg]

**A**

Chr1 (Chr7 in Fhet\_4.1)

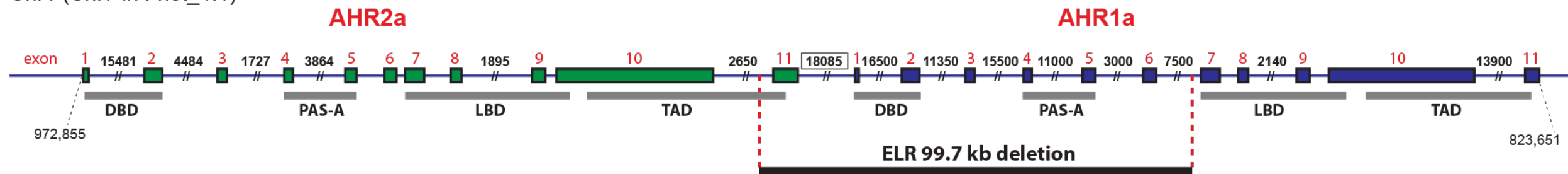

Chr18 (Chr24 in Fhet\_4.1)

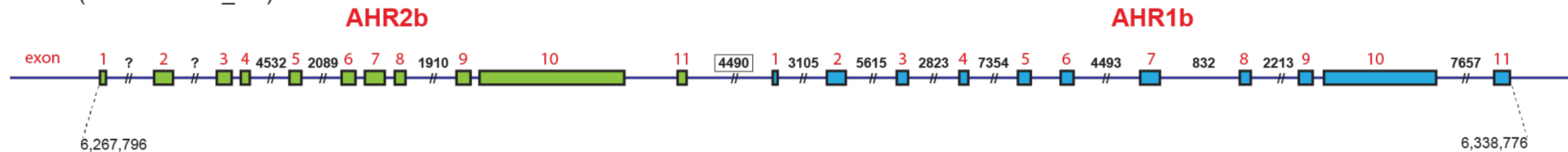**B**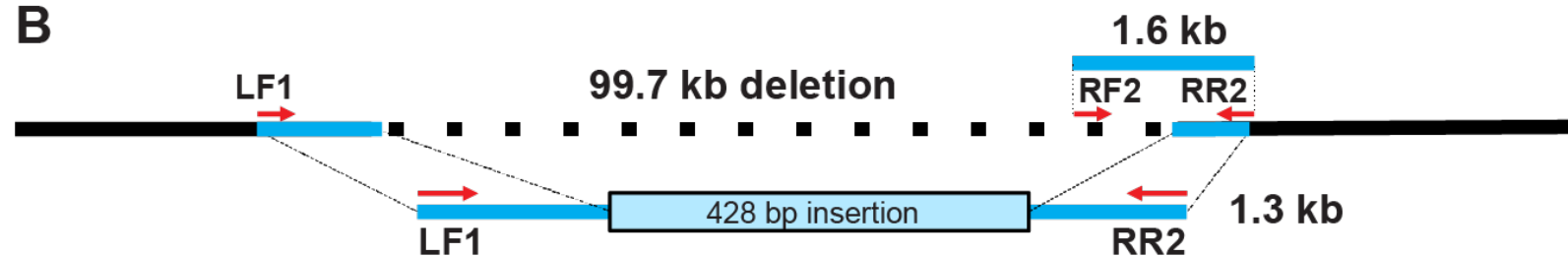

Supplement: Supplementary file 10 — Figure S7 [file EVA-17-e13648-s008.pdf]

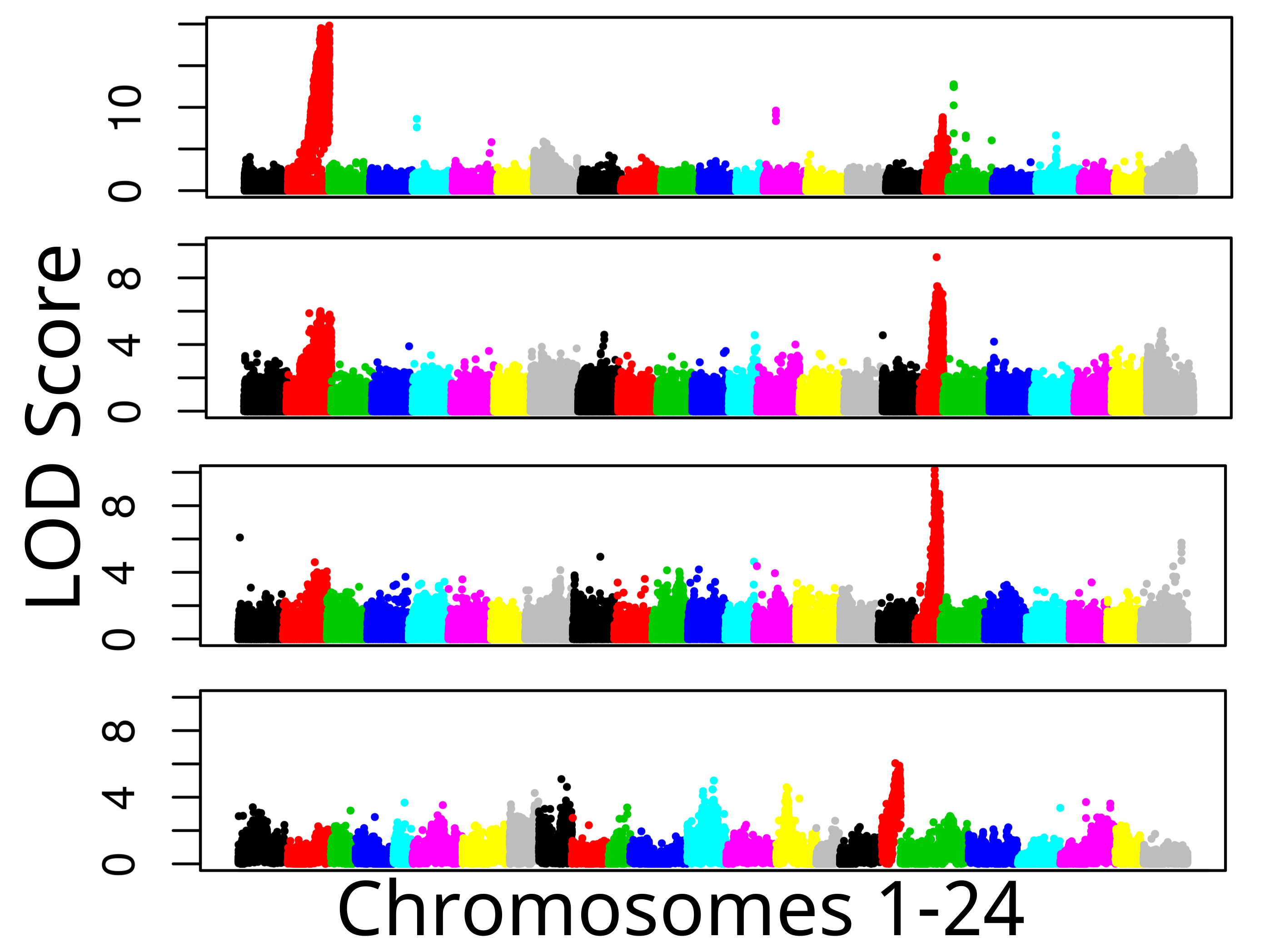

Supplement: Supplementary file 11 — Figure S8 [file EVA-17-e13648-s002.jpg]
