# Supplementary material for: Independently evolved pollution resistance in four killifish populations is largely explained by few variants of large effect
Source: Evol Appl. 2024 Jan 29;17(1):e13648. doi: 10.1111/eva.13648 (PMC10824703; doi:10.1111/eva.13648)

Metric MDS for Chromosome 5 unfiltered snps

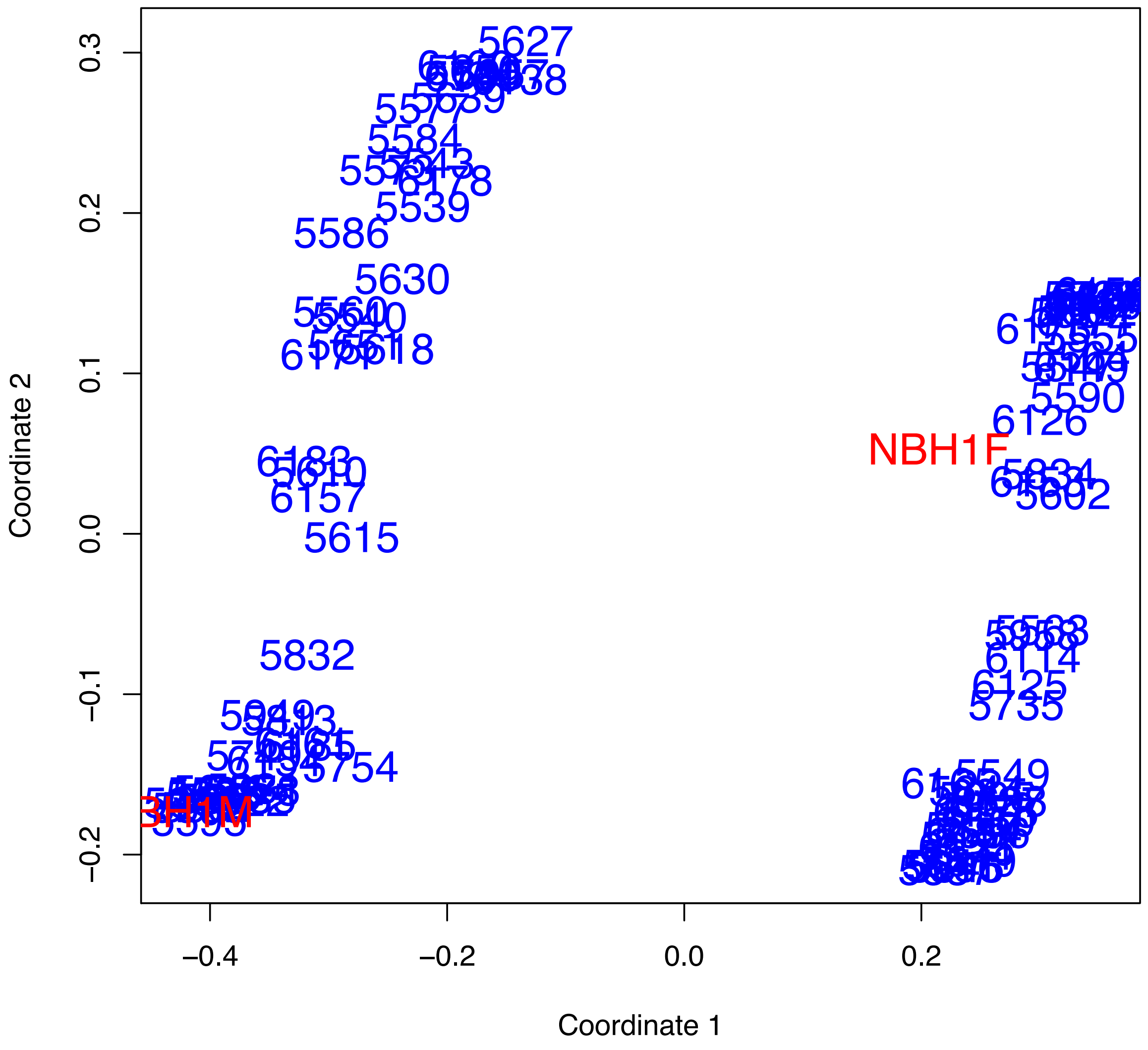

Metric MDS for Chromosome 4 unfiltered snps

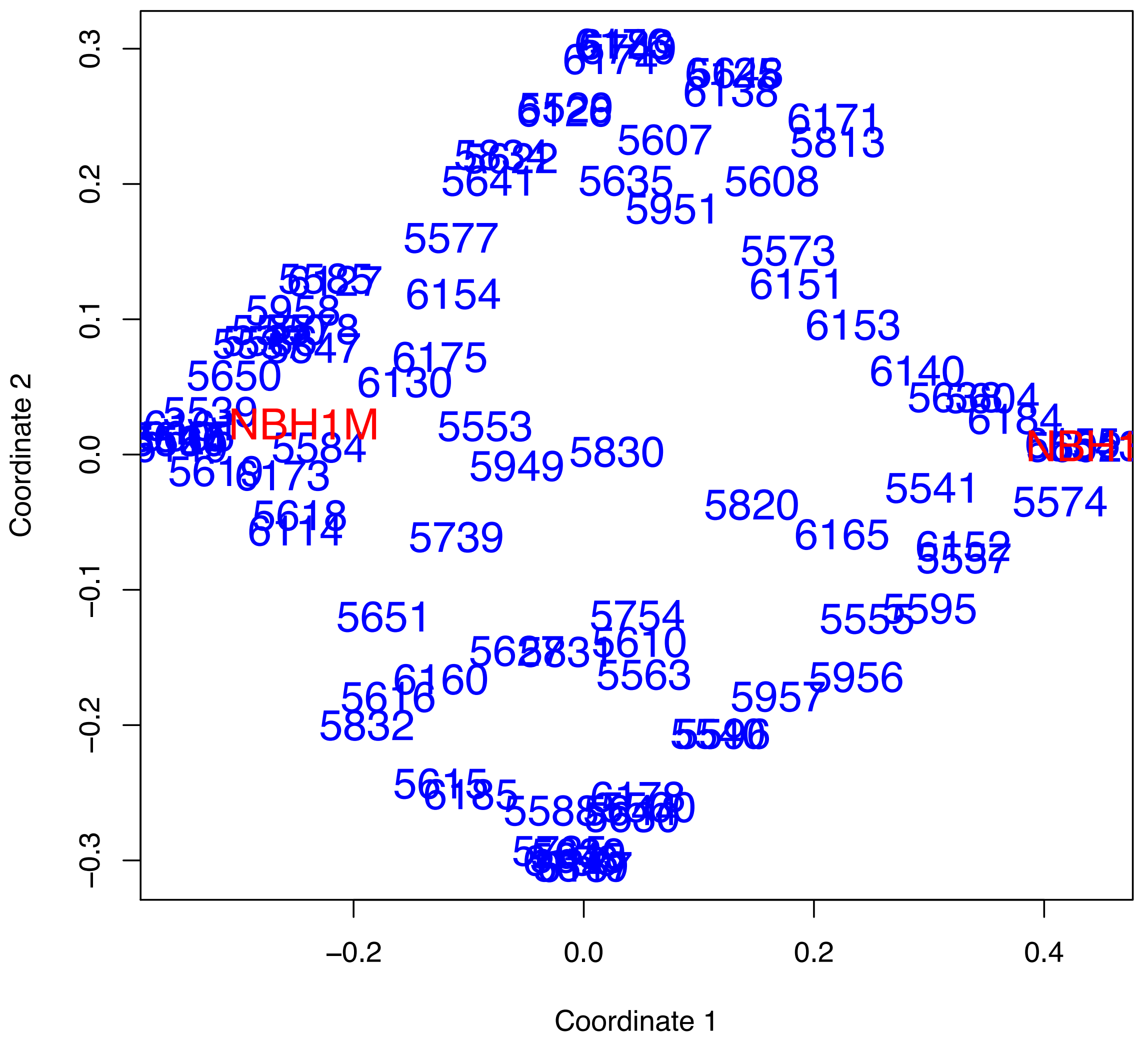

Supplement: Supplementary file 8 — Figure S5 [file EVA-17-e13648-s011.pdf]
