# Supplementary material for: Independently evolved pollution resistance in four killifish populations is largely explained by few variants of large effect
Source: Evol Appl. 2024 Jan 29;17(1):e13648. doi: 10.1111/eva.13648 (PMC10824703; doi:10.1111/eva.13648)
Supplement: Supplementary file 13 — Table S1 [file EVA-17-e13648-s009.docx]

Table S1. Field site identity, location, pollution levels, and PCB sensitivity level of local killifish populations.

| Field Site | Location | Latitude | Longitude | Site designation used in Reid et al 2016 | Sediment PCB concentration (ng/g dry weight) ^a^ | Log LC20, PCB-126 ng/L ^a^ |
| --- | --- | --- | --- | --- | --- | --- |
| NBH | New Bedford Harbor, MA, USA | 41.6676N | 70.9159W | T1 | 22,666 | 4.6 |
| BRP | Bridgeport Harbor, CT, USA | 41.1570N | 73.2189W | T2 | 1,700 | 4.1 |
| NEW | Newark, NJ, USA | 40.7006N | 74.1223W | T3 | 241 ^b^ | 4.1 |
| ELR | Atlantic Wood, Elizabeth River, Virginia, USA | 36.8078N | 76.2945W | T4 | 201 ^c^ | 5.3 |
| BI | Block Island, RI, USA | 41.1818N | 71.5793W | S1 | 3 | 1.4 |

^a^ Data are from: Nacci, D., M. Huber, D. Champlin, S. Jayaraman, S. Cohen, E. Gauger, A. Fong, and M. Gomez-Chiarri. 2009. Evolution of tolerance to PCBs and susceptibility to a bacterial pathogen (*Vibrio harveyi*) in Atlantic killifish (*Fundulus heteroclitus*) from New Bedford (MA, USA) harbor. Environ Pollut 157:857–864.

^b^ Although sediment PCB concentrations are lower at the NEW site relative to the most polluted sites, this site is also extremely polluted with highly toxic dioxins, which likely contributes to evolved cross-resistance to PCB-126: Crawford, D. W., N. L. Bonnevie, and R. J. Wenning. 1995. Sources of Pollution and Sediment Contamination in Newark Bay, New Jersey. Ecotoxicology and Environmental Safety 30:85–100; Wenning, R. J., M. A. Harris, B. Finley, D. J. Paustenbach, and H. Bedbury. 1993. Application of Pattern Recognition Techniques to Evaluate Polychlorinated Dibenzo-p-dioxin and Dibenzofuran Distributions in Surficial Sediments from the Lower Passaic River and Newark Bay. Ecotoxicology and Environmental Safety 25:103–125.

^c^ Although sediment PCB concentrations are lower at the ELR site relative to the most polluted sites, this site is also extremely polluted with highly toxic polycyclic aromatic hydrocarbons (PAHs), which likely contributes to evolved cross-resistance to PCB-126: Bieri, R. H., C. Hein, R. J. Huggett, P. Shou, H. Slone, C. Smith, and C.-W. Su. 1986. Polycyclic Aromatic Hydrocarbons in Surface Sediments from the Elizabeth River Subesturary. International Journal of Environmental Analytical Chemistry 26:97–113; Walker, S. E., R. M. Dickhut, and C. Chisholm-Brause. 2004. Polycyclic aromatic hydrocarbons in a highly industrialized urban estuary: Inventories and trends. Environ Toxicol Chem 23:2655–2664.

Table S2: Marker count per chromosome for each mapping family (NBH, BRP, NEW, and ELR), including the length of the chromosome (length in megabases), the number of SNP markers per chromosome (n) and the number of SNP markers per 1000 bases (n/kb).

|  | **NBH** | | | **BRP** | | | **NEW** | | | **ELR** | | |
| --- | --- | --- | --- | --- | --- | --- | --- | --- | --- | --- | --- | --- |
| chromosome | **Length (mbp)** | **n** | **n/kb** | **Length (mbp)** | **n** | **n/kb** | **Length (mbp)** | **n** | **n/kb** | **Length (mbp)** | **n** | **n/kb** |
| 1 | 39.2 | 2073 | 18.9 | 39.3 | 840 | 46.8 | 39.3 | 921 | 42.7 | 39.3 | 2985 | 13.2 |
| 2 | 37.9 | 1905 | 19.9 | 37.8 | 541 | 69.8 | 37.6 | 1131 | 33.3 | 37.9 | 2644 | 14.3 |
| 3 | 37.0 | 1441 | 25.7 | 37.0 | 754 | 49.1 | 37.0 | 786 | 47.1 | 37.0 | 1448 | 25.5 |
| 4 | 39.7 | 1442 | 27.5 | 39.3 | 834 | 47.1 | 40.0 | 730 | 54.8 | 40.0 | 2301 | 17.4 |
| 5 | 38.0 | 1241 | 30.6 | 37.8 | 675 | 56.0 | 38.0 | 628 | 60.5 | 38.0 | 1116 | 34.0 |
| 6 | 34.5 | 1027 | 33.6 | 34.3 | 738 | 46.5 | 34.5 | 1128 | 30.6 | 34.4 | 3062 | 11.2 |
| 7 | 38.1 | 1421 | 26.8 | 38.5 | 600 | 64.2 | 38.5 | 1364 | 28.2 | 38.5 | 2891 | 13.3 |
| 8 | 41.1 | 1473 | 27.9 | 41.1 | 588 | 70.0 | 41.1 | 903 | 45.5 | 41.1 | 1722 | 23.9 |
| 9 | 33.0 | 1048 | 31.5 | 32.9 | 679 | 48.5 | 32.9 | 1030 | 31.9 | 33.0 | 1987 | 16.6 |
| 10 | 34.5 | 1144 | 30.2 | 29.6 | 586 | 50.6 | 34.6 | 1511 | 22.9 | 34.6 | 2234 | 15.5 |
| 11 | 34.3 | 647 | 53.1 | 34.4 | 589 | 58.4 | 34.4 | 933 | 36.9 | 34.4 | 1181 | 29.1 |
| 12 | 34.6 | 1548 | 22.4 | 34.6 | 643 | 53.8 | 34.6 | 610 | 56.8 | 34.6 | 3516 | 9.8 |
| 13 | 25.5 | 1251 | 20.4 | 25.5 | 395 | 64.5 | 25.5 | 1052 | 24.2 | 26.1 | 2367 | 11.0 |
| 14 | 35.8 | 2021 | 17.7 | 35.8 | 834 | 42.9 | 35.8 | 1088 | 32.9 | 35.8 | 3053 | 11.7 |
| 15 | 37.6 | 2303 | 16.3 | 37.5 | 825 | 45.5 | 37.5 | 859 | 43.7 | 37.6 | 2424 | 15.5 |
| 16 | 32.3 | 1414 | 22.8 | 32.3 | 712 | 45.3 | 32.3 | 1532 | 21.1 | 32.3 | 1361 | 23.7 |
| 17 | 33.3 | 974 | 34.2 | 33.2 | 678 | 49.0 | 33.3 | 1347 | 24.8 | 33.3 | 2561 | 13.0 |
| 18 | 20.8 | 676 | 30.7 | 20.8 | 359 | 57.9 | 20.8 | 582 | 35.7 | 20.8 | 1127 | 18.4 |
| 19 | 42.5 | 2146 | 19.8 | 42.5 | 933 | 45.5 | 42.5 | 1541 | 27.6 | 42.4 | 4104 | 10.3 |
| 20 | 38.3 | 2725 | 14.1 | 38.3 | 821 | 46.7 | 38.3 | 2201 | 17.4 | 38.4 | 3036 | 12.7 |
| 21 | 40.3 | 1784 | 22.6 | 40.0 | 712 | 56.2 | 40.3 | 1341 | 30.1 | 40.3 | 2588 | 15.6 |
| 22 | 29.9 | 1607 | 18.6 | 29.9 | 807 | 37.0 | 29.8 | 1282 | 23.3 | 29.9 | 3393 | 8.8 |
| 23 | 30.6 | 2041 | 15.0 | 30.6 | 629 | 48.7 | 30.6 | 1494 | 20.5 | 30.6 | 1694 | 18.1 |
| 24 | 43.3 | 2352 | 18.4 | 43.7 | 953 | 45.8 | 43.7 | 1125 | 38.8 | 43.2 | 2694 | 16.0 |

Table S3. PCR-verified AHR1a/2a deletion genotype (del = AHRa deletion, wt= no deletion wild type) counts for resistant (phenotype score 0-1) and sensitive (phenotype score 4-5) embryos included in the QTL analysis from the ELR population. The deletion haplotype was segregating in our mapping family, but did not account for any variation in phenotype.

| Phenotype score | Genotype Count | | |
| --- | --- | --- | --- |
|  | wt/wt | wt/del | del/del |
| 0-1 | 10 | 22 | 8 |
| 4-5 | 16 | 25 | 6 |

Table S4. Mapping family genotype counts and estimated population frequency of the amino acid variant Trp🡪Leu at residue 224 in AIP.

| AIP (aa 224) | | | Count | | | Estimated pop. Frequency (Trp/Leu) | |
| --- | --- | --- | --- | --- | --- | --- | --- |
|  |  |  | Trp/Trp | Trp/Leu | Leu/Leu | Reference | Resistant |
| Phenotype score (0=resistant, 5=sensitive) | NBH | 0 | 0 | 0 | **4** | 1.00/0.00 | 0.26/0.74 |
|  |  | 5 | **2** | **2** | 0 |  |  |
|  | BRP | 0 | 0 | **2** | **2** | 0.97/0.03 | 0.30/0.70 |
|  |  | 5 | **2** | **2** | 0 |  |  |
|  | NEW | 0 | 0 | **2** | **1** | 0.96/0.04 | 0.28/0.72 |
|  |  | 5 | 0 | **1** | 0 |  |  |
|  | ELR | 0 | **4** | 0 | 0 | 1.00/0.00 | 1.00/0.00 |
|  |  | 5 | **3** | 0 | 0 |  |  |

Table S5. ELR mapping family genotype counts and estimated population frequency of the amino acid variant Ile🡪Val at residue 252 in AIP.

| AIP (aa 252) | | | Count | | | Estimated pop. Frequency (Ile/Val) | |
| --- | --- | --- | --- | --- | --- | --- | --- |
|  |  |  | Ile/Ile | Ile/Val | Val/Val | Reference | Resistant |
| Phenotype | ELR | 0 | **2** | **12** | **5** | 1.00/0.00 | 0.52/0.48 |
|  |  | 5 | **6** | **7** | **3** |  |  |

Table S6. Parameter estimates for QTLs under a binary phenotype using the Haley-Knott regression method in rQTL. Top table of models (All QTL Models) includes estimates for small effect QTL in the full multi-QTL model. Bottom table of models are estimates for each QTL including only the top two QTL in each family included in the full-model. Each QTL in the model is estimated with a dominant effect (d) and an additive effect (a).

| All QTL Models | | | | |
| --- | --- | --- | --- | --- |
| Cross | QTL | est | SE | t |
| NBH | Intercept | 0.6652 | 1.0846 | 0.613 |
|  | 2@38.2a | -7.0475 | 2.5121 | -2.805 |
|  | 2@38.2d | 3.7086 | 2.0607 | 1.8 |
|  | 8@54.7a | 3.2707 | 1.7192 | 1.902 |
|  | 8@54.7d | 0.1053 | 1.7152 | 0.061 |
|  | 18@31.8a | 4.854 | 2.1057 | 2.305 |
|  | 18@31.8d | 2.0388 | 2.077 | 0.982 |
| BRP | Intercept | -0.03512 | 0.36781 | -0.095 |
|  | 2@19.6a | 2.4007 | 0.63454 | 3.783 |
|  | 2@19.6d | 1.49404 | 0.74727 | 1.999 |
|  | 18@37.6a | -2.6215 | 0.57963 | -4.523 |
|  | 18@37.6d | 1.07479 | 0.71772 | 1.498 |
| NEW | Intercept | 0.7162 | 0.5047 | 1.419 |
|  | 2@45.0a | -2.6599 | 0.8686 | -3.062 |
|  | 2@45.0d | 0.3367 | 0.9413 | 0.358 |
|  | 11@47.7a | -0.4927 | 0.7169 | -0.687 |
|  | 11@47.7d | -3.2094 | 1.2606 | -2.546 |
|  | 18@49.8a | 3.7895 | 0.9103 | 4.163 |
|  | 18@49.8d | 2.175 | 1.1147 | 1.951 |
| ELR | Intercept | -0.4261 | 0.3661 | -1.164 |
|  | 18@37.1a | 2.1897 | 0.6041 | 3.624 |
|  | 18@37.1d | 0.1791 | 0.7327 | 0.245 |

| Top 2 QTL Models | | | | |
| --- | --- | --- | --- | --- |
| Cross | QTL | est | SE | t |
| NBH | Intercept | -1.982 | 46.132 | -0.043 |
|  | 2@38.2a | -18.606 | 160.777 | -0.116 |
|  | 2@38.2d | 6.113 | 160.782 | 0.038 |
|  | 18@25.1a | 12.695 | 131.678 | 0.096 |
|  | 18@25.1d | 1.744 | 131.685 | 0.013 |
| BRP | Intercept | -0.03512 | 0.36781 | -0.095 |
|  | 2@19.6a | 2.4007 | 0.63454 | 3.783 |
|  | 2@19.6d | 1.49404 | 0.74727 | 1.999 |
|  | 18@37.6a | -2.6215 | 0.57963 | -4.523 |
|  | 18@37.6d | 1.07479 | 0.71772 | 1.498 |
| NEW | Intercept | 0.5132 | 0.3896 | 1.317 |
|  | 2@45.0a | -2.8984 | 0.7844 | -3.695 |
|  | 2@45.0d | 0.5945 | 0.8774 | 0.678 |
|  | 18@49.8a | 3.4033 | 0.7935 | 4.289 |
|  | 18@49.8d | 1.2435 | 0.86 | 1.446 |
| ELR | Intercept | -0.4261 | 0.3661 | -1.164 |
|  | 18@37.1a | 2.1897 | 0.6041 | 3.624 |
|  | 18@37.1d | 0.1791 | 0.7327 | 0.245 |
